# Retracted: Single-Segment Lumbar Intervertebral Disc Nucleus Excision on the Stability of Lumbar Segmental Sagittal Plane

**DOI:** 10.1155/2022/9870917

**Published:** 2022-11-17

**Authors:** Computational and Mathematical Methods in Medicine

*Computational and Mathematical Methods in Medicine* has retracted the article titled “Single-Segment Lumbar Intervertebral Disc Nucleus Excision on the Stability of Lumbar Segmental Sagittal Plane” [1] due to concerns that the peer review process has been compromised.

Following an investigation conducted by the Hindawi Research Integrity team [2], significant concerns were identified with the peer reviewers assigned to this article; the investigation has concluded that the peer review process was compromised. We therefore can no longer trust the peer review process and the article is being retracted with the agreement of the Chief Editor.

The authors do not agree to the retraction; author Xiaobiao Du could not be reached by the publisher using the email address provided to the publisher with the article submission.
